# Research on Hot Spot Fault Detection Method Based on Infrared Images of Photovoltaic Modules in Complex Background

**DOI:** 10.3390/s26031024

**Published:** 2026-02-04

**Authors:** Lei Li, Weili Wu, Zhong Li

**Affiliations:** College of Electrical and Control Engineering, Xi’an University of Science and Technology, Xi’an 710054, China; xkd_ll286@163.com (L.L.); oldrifle@163.com (Z.L.)

**Keywords:** deep learning, fault inspection, hot-spot detection, image segmentation, YOLOv8

## Abstract

Aiming at the problem that fault characteristics cannot be effectively expressed due to the low pixel proportion of the hot spot target and background interference when detecting hot spot faults in complex environments, a photovoltaic module hot spot fault detection method integrating U-Net and YOLOv8 is proposed. Firstly, the U-Net segmentation network is introduced to remove pseudo-high-brightness heat sources in the background and highlight the contour features of the photovoltaic panels, laying a good foundation for the subsequent photovoltaic hot spot fault detection tasks. Secondly, a detection network is built based on the YOLOv8 framework. Aiming at the problems that it is difficult to extract the hot spot features of photovoltaic panels of different sizes and to balance the reasoning speed and detection accuracy, a detection network based on deformable convolution and GhostNet is designed. Furthermore, to enhance the adaptability of the convolutional neural network to multi-scale hot spot targets, deformable convolution (DCN) is introduced into the YOLOv8 network. By adaptively adjusting the shape and size of the receptive field, the detection accuracy is further improved. Then, aiming at the issue that it is difficult to balance accuracy and speed in the detection network, the C2f_Ghost module is designed to simplify the network parameters and improve the model inference speed. To verify the effectiveness of the algorithm, a comparison is made with SSD, YOLOv5, YOLOv7, and YOLOv8. The results show that the proposed algorithm can accurately detect hot spot faults, with an accuracy of up to 88.5%.

## 1. Introduction

As the core component for energy conversion in photovoltaic power stations, photovoltaic modules are prone to being covered and obscured by dust, bird droppings and other dirt due to long-term exposure outdoors, which causes local temperature rise [1,2,3]. The hot spot effect poses a serious threat to photovoltaic modules and their individual cells, which may cause a rapid increase in local temperature, thereby leading to a decline in module performance or damage. Under high-temperature conditions, it will also accelerate the oxidation rate of battery solder joints. In extreme cases, it may even cause a fire [4,5,6]. At present, hot spot detection technologies are mainly divided into two categories: electrical characteristics and image characteristics.

The detection method based on electrical characteristics mainly involves building circuits around the photovoltaic modules to collect electrical characteristic parameters (output current, voltage, power, etc.) and then analyzing the electrical characteristic information using mathematical models or machine learning to detect hot spots on the photovoltaic modules [7,8,9]. Although this method can accurately identify the fault status, it is difficult to determine the specific location of the faulty solar cell. Moreover, it requires the configuration of external circuits and sensors, which makes the operation complex and costly in large-scale photovoltaic power stations [10,11]. Reference [12] proposed comparing the changes of power, current and voltage parameters during normal and fault periods. Under normal circumstances, these parameters should show a certain degree of stability and regularity. However, when a malfunction occurs, these physical parameters will change abnormally, resulting in phenomena such as power drop, voltage distortion or current fluctuation. The detection method based on image characteristics mainly utilizes infrared images to reflect the temperature distribution of solar cells under different working conditions and realizes hot spot detection through computer vision technology. Compared with the detection method based on electrical characteristic monitoring, this approach is more efficient and does not require the establishment of any peripheral physical circuits, thereby avoiding any impact on photovoltaic modules. In recent years, an increasing number of scholars have begun to conduct research on image detection of hot spots. Reference [13] utilized Canny edge detection to pre-extract photovoltaic arrays that might have hot spots and then statistically analyzed the temperature histogram distribution of this area to achieve hot spot detection. Although it has relatively high detection accuracy, it requires manual separation of the photovoltaic array area from the ground background in advance, resulting in low real-time performance. Reference [14] identified photovoltaic arrays by performing threshold segmentation and variance calculation on temperature data and then detected abnormal photovoltaic modules by extracting the median temperature and histogram projection within the module area. However, the detection accuracy of this method is easily affected by the complex background of the actual operating photovoltaic power station. Reference [15] constructed a basic model on the photovoltaic electroluminescence image dataset using a lightweight convolution and shared the knowledge with the real infrared dataset, thereby significantly improving the algorithm accuracy. Reference [16] improved the YOLOv5 algorithm based on the defect features of infrared images of photovoltaic arrays. By using the K-means clustering algorithm to select the appropriate benchmark anchor box, three types of hot spot detection with different shapes were established on the large-sized feature map generated through fusion. Reference [17] optimized the feature extraction network based on YOLOv5 using the lightweight focus structure and ShuffleNetv2 structure, thereby improving the hot spot detection accuracy.

To provide a broader context on industrial fault detection, recent advancements in multimodal learning have shown great potential. CNC-VLM [18] proposed an RLHF-optimized industrial large vision-language model for imbalanced CNC fault detection, demonstrating the efficacy of combining visual and linguistic features.

However, in the specific domain of PV hot-spot detection, the primary challenge remains the intrinsic thermal similarity between faults and background noise. The characteristics of infrared thermal imaging will cause the background area to be highlighted under excessive lighting conditions, making it difficult for the detection network to distinguish between foreground hot spot targets and background interference features, thereby limiting detection performance and increasing the risk of false detections. To address this specific problem, this paper proposes a targeted “segmentation first, detection later” strategy. Unlike generic model combinations, this design utilizes the segmentation stage as a spatial filter to physically exclude non-panel heat sources in the background, thereby enabling the subsequent network to accurately detect the hot spot problem. The main innovations and contributions are as follows:

(1) To avoid the difficulty of accurately locating the hot spot fault target due to the interference of background pseudo-highlight features, a U-Net segmentation network is designed to remove the complex background and highlight the contour features of the photovoltaic module, laying a good foundation for the subsequent detection tasks.
(2) To address the problems of difficulty in extracting multi-scale hot spot features and the balance between inference speed and detection accuracy, a deformable convolution and GhostNet is designed.
(3) To enhance the adaptability of convolutional networks to multi-scale hot spot targets, a deformable convolution (DCN) is introduced into the detection network. By adaptively adjusting the shape and size of the receptive field, the detection accuracy of multi-scale hot spot targets is improved.
(4) Aiming at the issue that it is difficult to balance accuracy and speed in the detection network, the C2f_Ghost module is designed to simplify the network parameters and improve the model reasoning speed.

## 2. Problem Settings

### 2.1. The Formation Mechanism of Hot Spots

**Figure 1:**
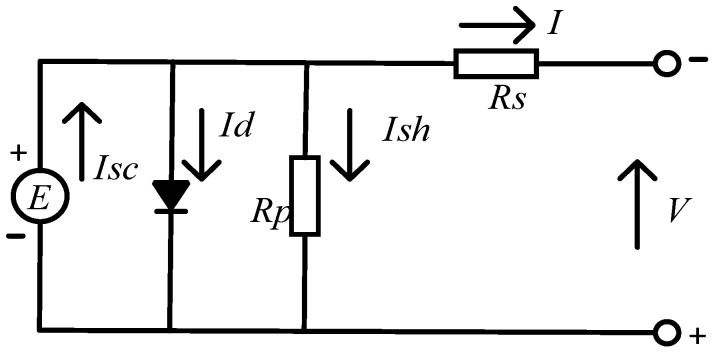
Equivalent circuit diagram of photovoltaic power generation.

The core component of a photovoltaic system is the photovoltaic cell, which can generate electricity from solar energy. This conversion process is accomplished through the photoelectric effect. When the intensity of sunlight increases, the photovoltaic cells will generate current, which flows into the external designed circuit to complete photovoltaic power generation [18,19]. The equivalent photovoltaic cell circuit diagram shown in Figure 1 demonstrates the basic principle of its power generation.

When the photovoltaic cells are operating under normal conditions, the currents at each branch satisfy the following Equation (1):

(1) $$I_{sc}=I_{d}+I_{sh}+I$$

In Equation (1), current source *E* generates photogenerated current *I_SC_*. Meanwhile, the photogenerated current *I_SC_* is also affected by the external working environment (such as the working environment temperature, light intensity and other conditions). *I_d_* is the current flowing through the diode branch, which is opposite in direction to the photogenerated current *I_SC_*. *R_P_* represents the equivalent parallel resistance of photovoltaic cells, which reflects the equivalent resistance due to factors such as their own defects. The physical meaning of *R_S_* is the internal resistance caused by the battery cell. *I* represents the output current of the photovoltaic cell for power generation.

When the photovoltaic cells are not obstructed in any way and are fully exposed to sunlight, operating in a normal state, the photovoltaic power generation current satisfies Equation (1), reaching the ideal state. However, in actual operation, photovoltaic power stations are often disturbed by various uncertain factors, such as sand and dust, leaves and bird droppings. The emergence of uncertain factors blocks the sunlight exposure of photovoltaic cells. These obstructions will cast shadows on the photovoltaic panels, reducing the amount of light radiation in the shaded areas, thus lowering the power generation of these areas, which is lower than that of other photovoltaic cells. Therefore, the overall power generation capacity of photovoltaic power is weakened, and the photogenerated current will also decrease, unable to produce a high and sufficient current, thus disrupting the normal working state of photovoltaic cells. At this point, the left side of Equation (1) is less than the right side. Due to the semiconductor nature of the solar cell, the photovoltaic cell undergoes reverse bias, causing a sharp increase in the local temperature of the photovoltaic module and generating hot spots. Such a situation not only may reduce the power generation capacity of photovoltaic power stations but in extreme cases, it may also lead to fires, causing significant economic losses to photovoltaic power stations. At the same time, photovoltaic power stations also have very fragile equipment. Once problems occur, they are very easily damaged. Therefore, it is of great significance to detect hot spots that may occur during the photovoltaic power generation process, and this step is an important part of the photovoltaic power generation system. When inspecting photovoltaic power stations, it is a basic requirement for photovoltaic power generation systems to maintain normal and stable operation to detect the appearance of photovoltaic hot spots as early as possible and take timely measures to troubleshoot.

### 2.2. The Difficulties in Hot Spot Detection

Most rooftop photovoltaic power stations on buildings are distributed photovoltaic power stations. The layout of such power stations is characterized by dispersion and diversity, and the installation of photovoltaic panels is not centralized. Meanwhile, due to the presence of a large number of structurally complex building materials around its working environment, their temperatures under sunlight are similar to those of photovoltaic strings, which may lead to incorrect detection of photovoltaic hot spots and cause significant interference [20,21].

**Figure 2:**
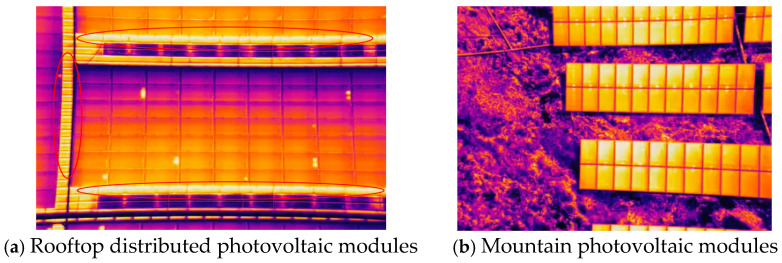
Infrared images of different photovoltaic power stations.

Figure 2a shows the interference brought by roof building materials to the detection of photovoltaic hot spots due to similar temperatures. The working environment of mountainous photovoltaic power stations is characterized by complex geographical conditions and rugged terrain and is influenced by various natural factors. Due to the interference of light factors, different types of solar power stations may all be affected. In infrared images, this interference has a high degree of similarity to the characteristics of hot spots, presenting as highlights, which may lead to misjudgment when detecting hot spots, as shown in Figure 2b.

In the working environment of photovoltaic modules, there exist objects with temperatures similar to those of the photovoltaic modules. In infrared temperature images, these objects have similar features to photovoltaic hot spots and are prone to confusion, which may lead to incorrect detection. To reduce the false detection rate of hot spots, it is necessary to analyze the interfering factors from multiple aspects and take corresponding measures to eliminate them. Light, current collectors and the background of the ground are all key interfering elements. However, the uneven distribution of solar cells leads to significant differences in the intensity of sunlight at different locations. In addition to the aforementioned interfering elements, the inspection speed of the unmanned aerial vehicle (UAV) and the inherent defects of the infrared camera lens may also cause the infrared images of the photovoltaic string to become blurred, which further affects the detection effect of hot spot faults.

## 3. Method

### 3.1. Overall Design of the Detection Algorithm

**Figure 3:**
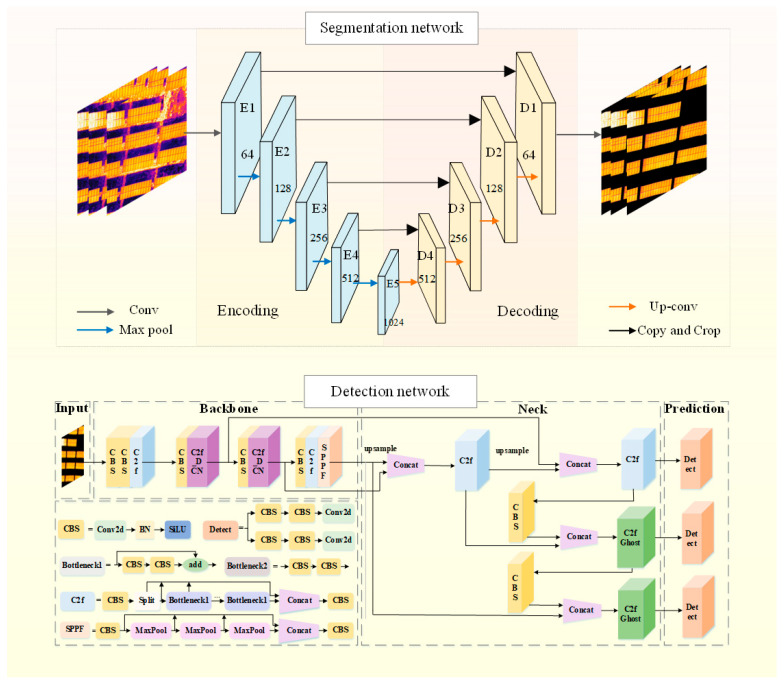
Overall block diagram of the proposed algorithm.

As shown in Figure 3, a photovoltaic module hot spot fault detection algorithm integrating U-Net and YOLOv8 is proposed. Firstly, input the infrared photovoltaic panel image into the U-Net network for segmentation to eliminate the background pseudo-highlight features. Based on the removal of background interference, the segmented images are sent to the YOLOv8 detection framework for the detection of photovoltaic panel defects. In this way, the situation of false detection can be dealt with efficiently, thereby enhancing the detection accuracy.

### 3.2. Photovoltaic Module Area Segmentation Network Based on U-Net

**Figure 4:**
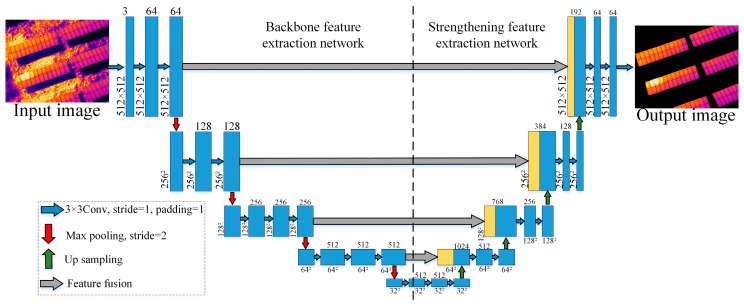
Block diagram of U-Net network structure.

As shown in Figure 4, the infrastructure of U-Net is divided into encoders and decoders, which correspond respectively to the extraction networks of backbone features and enhanced features. In the encoder section, the network gradually extracts image features through five concatenated convolutional layers, with the size of the convolutional kernels increasing successively to 64, 128, 256, and 512. At the same time, a 2 × 2 Max pooling operation is combined to reduce the size of the feature map and capture spatial information of different scales [22]. In terms of the activation function, U-Net selects the ReLU function to introduce nonlinear characteristics and enhance the fitting ability of the model. In the decoding section, the restoration of the original size and details of the image is achieved through stepwise upsampling and convolutional layers, while increasing the number of channels to meet different segmentation requirements. Feature fusion is carried out between the encoder and the decoder through skip connections. This mechanism helps preserve the detailed information of the image and improve the segmentation accuracy. Ultimately, U-Net uses a 1 × 1 × C convolution kernel to transform the fused feature map and outputs the segmentation result corresponding to the number of image segmentation categories.

### 3.3. Detection Network Based on YOLOv8

**Figure 5:**
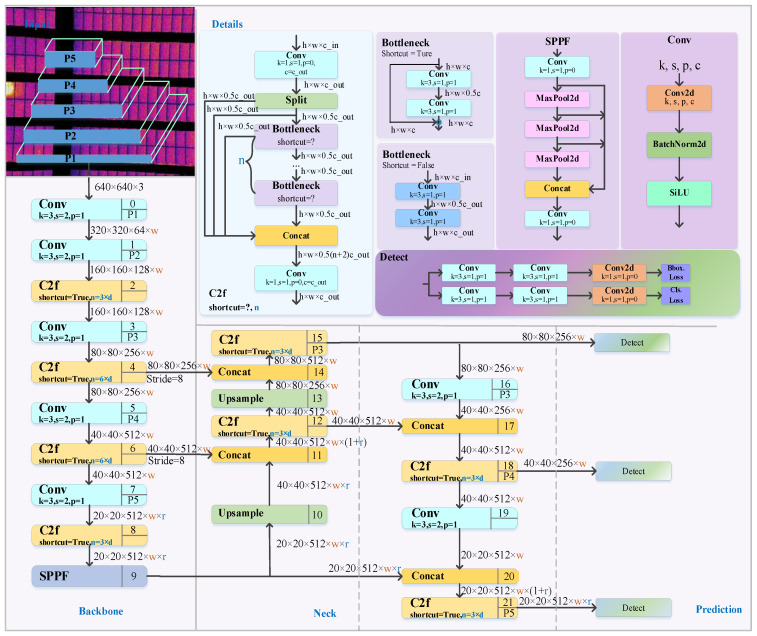
Schematic diagram of the YOLOv8 detection algorithm.

YOLOv8 includes YOLOv8-n, s, m, l, and x [23]. As the depth and width gradually expand, the parameters and computing resources required by the model also increase accordingly. Therefore, how to select appropriate model parameters has become a very important issue. By analyzing the hot spot data and comparing it, it was found that the YOLOv8n network is more suitable for the training of the dataset in this study, with a smaller model size and higher accuracy. The detection network mainly consists of four core parts, as shown in Figure 5.

In the Backbone part, the key modules consist of Conv, C2f (CSPDarknet53 to 2-Stage Feature Pyramid Network) and SPPF (Spatial Pyramid Pooling Faster). The Conv module is responsible for performing convolution operations. Combined with Batch Normalization and SiLU activation functions, it is used to extract and transform image features. Meanwhile, YOLOv8n adopts a brand new C2f structure, which effectively retains the gradient flow information in the image and thus achieves the effect of image lightweighting. The SPPF module based on spatial pyramid pooling can convert feature maps of different sizes into feature vectors of fixed sizes to support subsequent object detection and classification tasks.

The Neck network, as a key component connecting the Backbone network and the Head network, undertakes the important tasks of feature fusion and processing. Its characteristics are mainly reflected in its efficient multi-scale feature integration capability. It adopts a combined architecture of the Feature Pyramid Network (FPN) and the Path Aggregation Network (PAN) to achieve effective fusion of features of different scales. The FPN extracts features through the CNN and builds a pyramid, fusing multi-level features to enhance model performance, but it may lose target position information. To make up for this deficiency, the Neck network introduces the Path Aggregation Network (PAN). The PAN adopts a bottom-up structure. Through layer-by-layer upsampling and feature map fusion, it organically combines the low-level features with the high-level features, thereby accurately retaining spatial information. This fusion of context information enables the model to capture the details of the target and its contextual relationship with the surrounding environment more comprehensively, thereby significantly improving the accuracy of target detection.

In the prediction stage, the Head can accurately predict the types of target objects of different sizes by processing feature maps of various sizes and simultaneously obtain the precise position information of these target objects in the image. This mechanism ensures the efficiency and accuracy of the object detection task.

#### 3.3.1. Backbone Network

**Figure 6:**
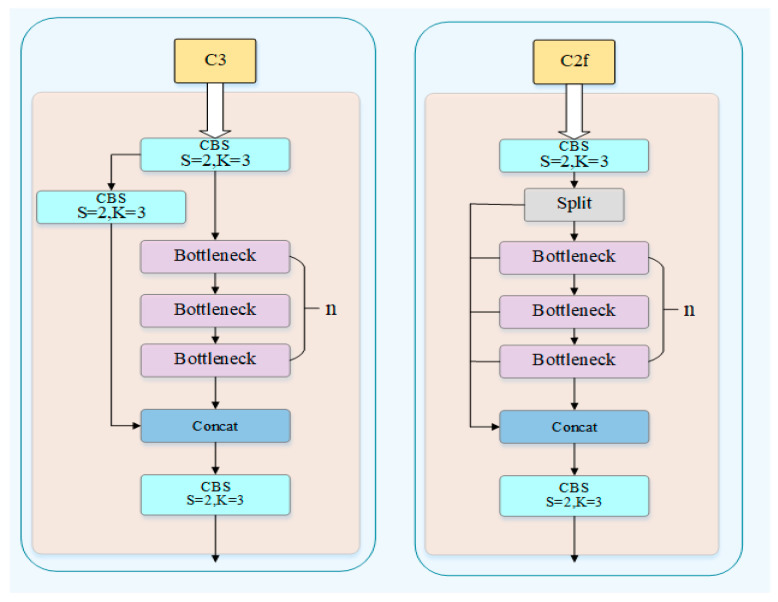
Schematic diagrams of the C2f module and the C3 module.

The C2f module provides a richer information flow path for gradient backhaul by adding more tributary paths, which helps the model to learn and optimize more effectively during the training process. The design of the C2f module enables the model to propagate error signals more effectively during the training process, thereby accelerating the convergence of the model and improving its performance. Therefore, this key difference between the C2f module and the C3 module provides important support for YOLOv8 to achieve excellent performance in object detection tasks. The following is the network structure diagram of the corresponding C2f and C3 modules in YOLOv8 and YOLOv5, as shown in Figure 6.

#### 3.3.2. FPN–PAN Structure

**Figure 7:**
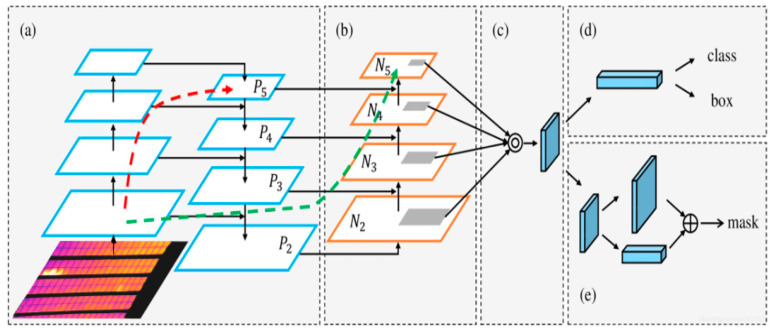
The connection layer structure combining FPN and PAN. (**a**) FPN backbone. (**b**) Bottom-up path augmentation. (**c**) Adaptive feature pooling. (**d**) Box branch. (**e**) Fully-connected fusion.

The connection layer adopts the PAN–FPN structure, including the Feature Pyramid Network (FPN) and the Path Aggregation Network (PANet). The Feature Pyramid Network is a feature extraction network used for object detection tasks. It adopts a top-down structure and conveys deep features through upsampling and shallow feature fusion. Unlike traditional feature fusion networks, the FPN predicts deep and shallow information on feature maps at different levels, respectively, and fuses them to obtain new feature maps. However, the FPN has obvious disadvantages: it only performs feature fusion between adjacent layers and lacks cross-layer information exchange. Although it enhances the deep semantic information, the shallow positioning information is ignored. As a supplement to the FPN, the path aggregation network introduces a bottom-up downsampling structure to transfer shallow positioning features and retains the high-level semantic information transfer mechanism in the FPN. Ultimately, the features of the two types are fused to achieve accurate prediction on images of different scales and enrich the information related to surface semantics and depth semantics contained in each dimension. The connection layer structure combining the FPN and PAN is shown in Figure 7.

#### 3.3.3. Detection Head

**Figure 8:**
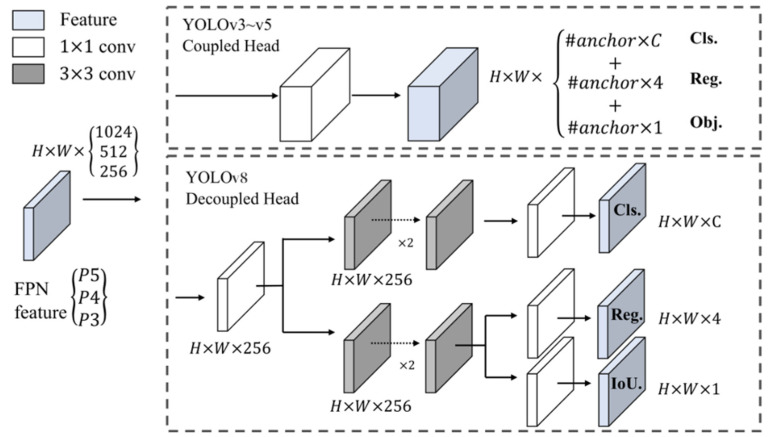
The structure diagram of the detection head.

In the detection head algorithm of YOLOv8, there are three key components, namely, classification (Cls), regression (Reg), and object detection (Obj). Cls is responsible for predicting the object category represented by each feature point and providing the corresponding category score. Reg determines the regression parameters of feature points to fine-tune the coordinates (x, y, w, h) of the target box and generate a more accurate prediction box. Obj distinguishes the foreground from the background by determining whether the feature points contain objects, reducing false detections and missed detections. The structure of the detection head is shown in Figure 8.

### 3.4. Hot Spot Detection Network Based on DCN-GYOLOv8

#### 3.4.1. Deformable Convolution Network

**Figure 9:**
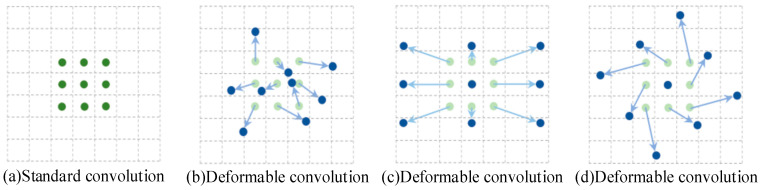
Schematic diagram of DCN.

The algorithm design goal of the Deformable Convolution Network (DCN) [24] is to improve the stability of the model when learning complex targets. Because convolutional neural networks can automatically obtain the relationships between pixels within the region of interest from a large number of image and video sequence samples, they can better adapt to the size of the target object during detection. In DCN technology, the shape of the convolution kernel has transcended the simple rectangle. The construction of this convolution kernel demonstrates remarkable universality and flexibility. It can adapt to the requirements of different network stages, diverse feature maps, and various pixel points, thus potentially achieving the optimal convolution effect in all situations. This feature ensures that the network can fully extract and utilize the key information in images when handling complex tasks, thereby enhancing overall performance. When these images are input into the convolutional neural network, a large amount of redundant information is generated, which reduces the model’s performance. The principle of the DCN is shown in Figure 9.

**Figure 10:**
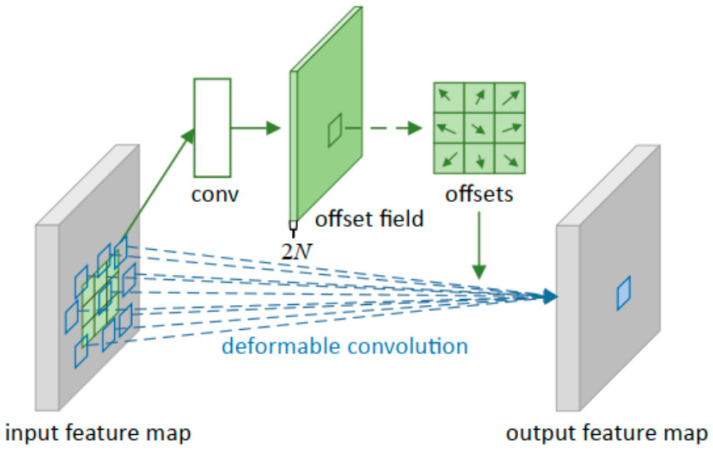
The structure diagram of the DCN module.

The core idea of deformable convolution is to obtain more refined spatial transformation information by applying convolution operations on the input feature map and introducing learnable offsets. Specifically, at each pixel position of the input feature map, flexible adjustment of the standard convolution kernel is achieved by learning a set of offsets (x, y). This learning of offsets is part of the convolution operation, aiming to capture geometric transformations and deformations in the image. For different channels of the same feature map, deformable convolution adopts the same prediction offset to ensure the consistency and efficiency of cross-channel information. This model can handle the output information when there is noise or texture change in the image and can effectively improve the accuracy of image recognition and classification. The structure of the DCN module is shown in Figure 10.

The traditional convolution calculation method first selects a group of pixel points from the input feature map and then uses convolution technology to analyze these sampled data, thereby obtaining the final calculation result.

(2) $$y(p_{0})=\sum_{p_{n\in R}}w\left(p_{n}\right)\cdot x(p_{0}+p_{n})$$

For deformable convolution, $\;\left\{\Delta p_{n}|n\;=\;1,\;2,\cdots ,\;N\right\}$. The following formula shows the calculation process of deformable convolution:

(3) $$y(p_{0})=\sum_{p_{n\in R}}w\left(p_{n}\right)\cdot x(p_{0}+p_{n}+\Delta p_{n})$$

The offset calculated by the convolution method above is a decimal, so sample collection cannot be performed directly. Therefore, the DCN chose bilinear interpolation as its sampling method to achieve the offset effect, and the mathematical formula above is precisely a description of the bilinear interpolation process.

When performing the object detection task, the bounding box clarifies the positioning of the target by the object detector at different stages. The traditional method uses boundary points to represent bounding boxes and obtains the target by matching them. Although the calculation of bounding boxes is relatively simple, they only provide a rough positioning of the target and cannot fully simulate the shape and posture of the target. In addition, due to the lack of consideration of the characteristics of the images themselves, these bounding boxes are not robust to moving targets in some complex situations. In the object detection task, these cells may simultaneously contain information about the background region and the foreground target, so the extracted features may be interfered with by significantly inaccurate information. This kind of interference may stem from noise in the background area or partial occlusion of the foreground target, thereby reducing the validity and reliability of the extracted features. This decline in feature quality will inevitably affect the classification ability of the object detection algorithm, which may lead to incorrect classification results or performance degradation. Therefore, when designing and implementing object detection algorithms, special attention should be paid to how to handle this inaccurate information from background or foreground regions to ensure the accuracy of feature extraction and the stability of classification performance.

This study incorporated the DCN module into the backbone network of YOLOv8s, mainly based on the following three considerations. Firstly, the weights of traditional convolutional kernels are fixed, which leads to the fact that the size of the receptive field remains consistent when processing different image position regions. However, in practical applications, objects usually exhibit different scales and deformations, and these changes are reflected as different positional features on the feature map. To accurately capture these changes, the network needs to have the ability to adaptively adjust the receptive field so as to better meet the detection requirements of objects of different scales or deformations. Deformable convolution introduces additional offsets, enabling the convolution kernel to deform according to the actual size and shape of the object during sampling, thereby achieving adaptive adjustment of the receptive field and endowing the network with higher robustness. In contrast, traditional convolution operations often rely on a fixed grid during sampling and cannot be adjusted according to the specific shape of the object. This may lead to a distorted description of the object’s shape and thereby affect the detection performance. Deformable convolution can better capture the detailed information of objects and improve the accuracy of detection by dynamically adjusting the sampling position. Finally, for small targets, due to their small size and irregular shape, traditional convolution operations often struggle to effectively extract their feature information, resulting in poor detection performance. The DCN module can better adapt to the size and shape changes of small targets by adaptively adjusting the receptive field. In the YOLOv8 network, the main function of the backbone is to extract the features of the input image. Introducing a DCN can significantly enhance the accuracy of feature extraction, enabling the model to capture the key features of objects more accurately, and, at the same time, enhance the robustness of the model to ensure it maintains stable performance when dealing with complex and variable input data.

#### 3.4.2. GhostNet Module

**Figure 11:**
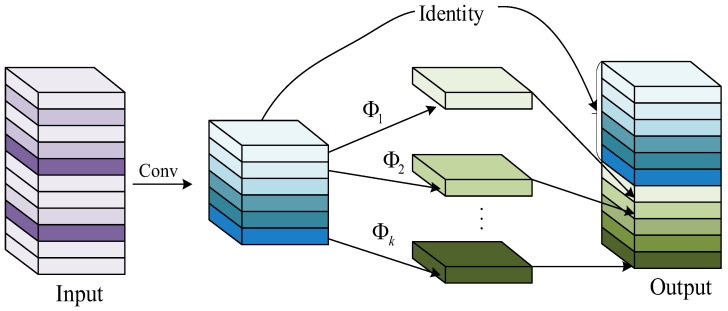
The core design of GhostNet.

The core of GhostNet lies in its unique GhostConv module, as shown in Figure 11. This module innovatively replaces the traditional convolution operation, significantly reducing the computational burden and parameter scale of the network. Under the premise of ensuring the size and number of channels of the convolutional output feature map are unchanged, GhostNet has achieved higher computational efficiency and lower resource consumption by optimizing the network structure. When building the network, GhostNet first uses several standard convolution kernels for initial information extraction and then employs efficient linear transformation in the GhostConv module to further process the feature map. Ultimately, the feature maps from different stages are combined through the Concat operation to form a complete feature representation. As a lightweight network architecture, GhostNet not only enhances the operational efficiency of the model but also provides strong support for the application of deep learning in resource-constrained scenarios such as mobile devices and embedded systems.

**Figure 12:**
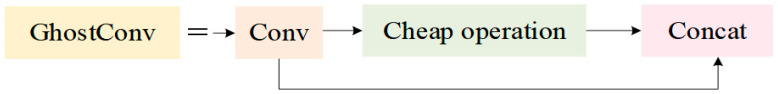
GhostConv structure.

As shown in Figure 12, in this process, “Cheap operation” represents a cost-effective linear calculation method [25,26]. GhostConv first performs convolution using half the size of the original convolution kernel, thereby generating partial feature maps. By simplifying the calculation steps with a 5 × 5 size and a 1 step size, we obtained the additional feature map. On this basis, further processing is carried out on these newly generated feature maps to obtain more effective information. Then, we used the Concat operation to fuse these two sets of feature maps into the final output result.

**Figure 13:**
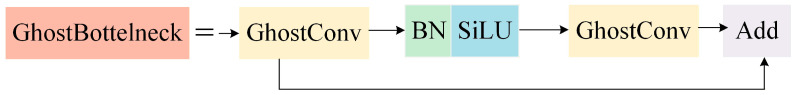
GhostBottelneck structure.

As shown in Figure 13, GhostBottelneck first uses GhostConv in the extension layer to increase the number of channels of the input feature map, enriching the feature representation and enhancing the ability to recognize complex patterns. Then, the feature map is processed through regularization and SiLU activation function to enhance the generalization ability and nonlinear feature expression ability of the model. Subsequently, the second GhostConv layer reduces the number of channels in the output feature map to match the input, maintaining the coherence of the network structure. Ultimately, feature fusion is achieved through residual join to enhance the model’s representational and learning capabilities.

**Figure 14:**
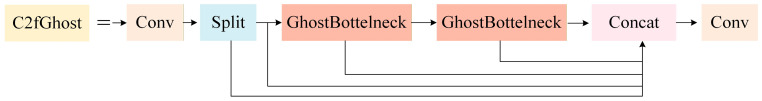
C2fGhost structure.

This study designed a lightweight prediction head of C2f_Ghost, as shown in Figure 14. By optimizing the traditional network architecture, GhostBottleneck is used to replace the Bottleneck part of C2f. By implementing cross-stage feature fusion technology and truncated gradient flow strategy, not only has the flexibility and efficiency of feature learning between network levels been enhanced but the possible negative impact of redundant gradient information on network learning performance has also been significantly reduced. This method can effectively improve the classification ability of traditional deep neural network models for datasets of different scales and achieve a high accuracy rate. In addition, by integrating the GhostConv and C2fGhost modules, the use of 3 × 3 convolution operations, which are widely applied in traditional networks, has been significantly reduced, thereby effectively decreasing the volume of the network model, the number of parameters, and the computational requirements. These improvements have made the application of the network in photovoltaic hot spot fault detection more efficient and feasible.

## 4. Experimental Verification and Result Analysis

### 4.1. Acquisition and Processing of Datasets

**Figure 15:**
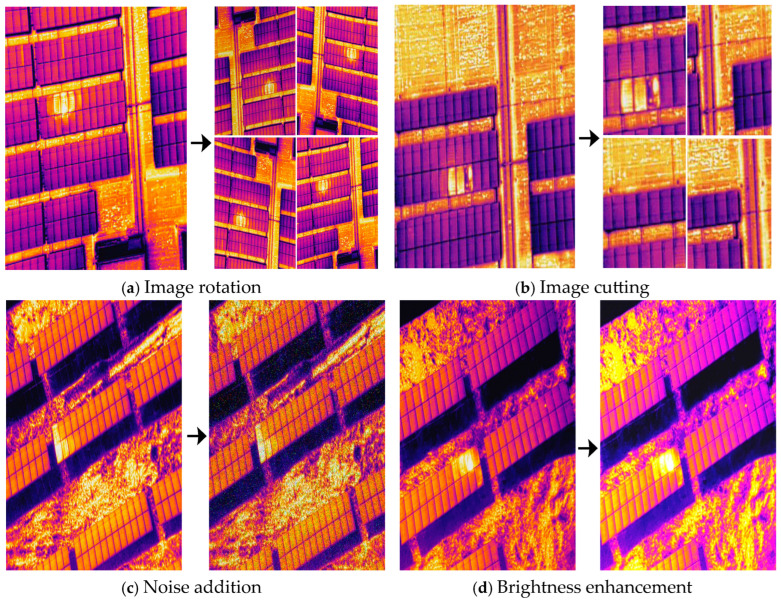
Data augmentation effect diagram.

The infrared image dataset used in this article was captured by a drone above an actual operating photovoltaic power station. To expand the richness of the samples and improve the generalization ability of the network, a series of data augmentation techniques were adopted to preprocess the data set. These techniques include image rotation and cropping operations, as well as methods such as injecting noise into the image and adjusting its brightness. By implementing these operations, the sample data was successfully expanded, thereby constructing a more complete and diverse dataset, providing richer data support for subsequent model training, as shown in Figure 15.

The final dataset consists of 1200 images, covering three distinct scenes: rooftop distributed stations (500 images), mountain centralized stations (400 images), and complex lighting scenarios (300 images). These are split into training, validation, and testing sets in a 6:2:2 ratio (720:240:240 images).

### 4.2. Experimental Environment Configuration

For ground truth generation, we employed the LabelMe annotation tool. A two-round quality control procedure was implemented: initially, researchers manually annotated the PV panel masks and hot-spot bounding boxes. Subsequently, cross-validation was performed by a senior expert to correct edge inaccuracies and label errors, ensuring the reliability of the segmentation masks and detection boxes. During the training phase of the model, preprocessing is carried out first, that is, the resolution of the input image is uniformly standardized to 640 × 640 pixels to ensure that the data received by the model has a consistent spatial scale. Subsequently, an asynchronous stochastic gradient descent algorithm with a momentum term of 0.936 was adopted for the optimization training of the model. This choice aims to accelerate convergence and stabilize the training process by introducing a momentum term. Meanwhile, the asynchronous training method can effectively utilize computing resources and enhance training efficiency. In each iteration, we set each batch of training data to contain 16 images and sent them into the network in 48 Epochs in sequence for training to ensure that the model could fully learn the features and patterns in the dataset. To better optimize the training process, a learning rate adjustment scheme was set: in the first 200 rounds of iterations, the learning rate was set to 0.01 to accelerate the convergence speed. As the number of iterations increased, the learning rate was gradually reduced to 0.001 in the next 100 rounds of iterations to facilitate more precise adjustments to the model parameters. The configuration of the experimental platform are shown in Table 1, and the experimental hyperparameter settings are shown in Table 2.

### 4.3. Evaluation Metrics

To comprehensively and objectively evaluate the performance of the proposed hot-spot detection algorithm, four standard quantitative indicators were adopted: Precision (P), Recall (R), and mean Average Precision at IoU = 0.5 (mAP@0.5). Precision reflects the model’s ability to distinguish negative samples. Recall measures the model’s ability to locate all positive samples. Furthermore, mAP@0.5 is utilized to evaluate the overall detection accuracy across different confidence thresholds. It is calculated by averaging the Average Precision (AP) of all classes with an Intersection over Union (IoU) threshold set to 0.5, serving as a robust metric for assessing the global performance of the detection network.

### 4.4. Analysis of Experimental Results

**Figure 16:**
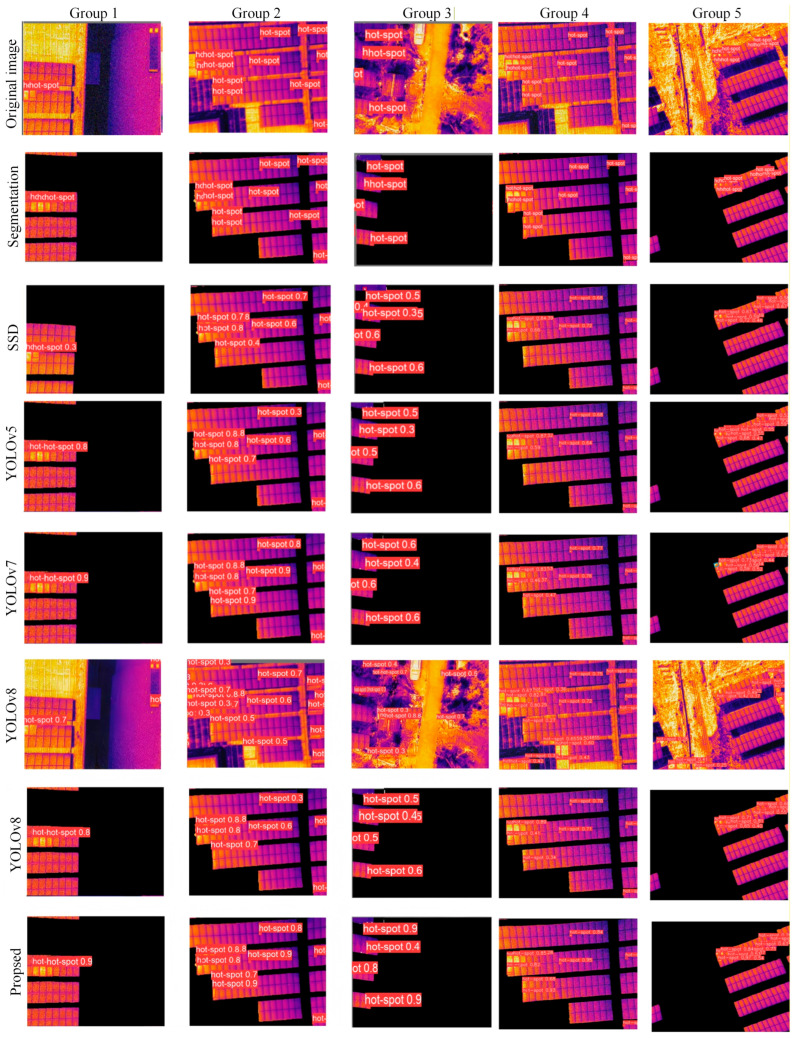
Comparison of test results.

Five scenarios with fewer hot spot faults, multi-object hot spot faults, small hot spot faults, low-contrast small hot spot faults and complex background environments were selected for experimental testing. In this study, an unsegmented detection algorithm and other classic object detection algorithms (SSD, YOLOv5, and YOLOv7) were used to train and detect the dataset and conduct a comparative analysis with the algorithm used in this paper. The result comparison is shown in Figure 16.

By analyzing the detection of hot spots with a small number of faults in Group 1 in Figure 16, it can be seen that the SSD algorithm has a problem of missed detection, and the original unsegmented image has a problem of false detection. The detection performance of the other algorithms is good. By analyzing the hot spot detection under the multi-target fault of Group 2 in Figure 16, it can be seen that the SSD, YOLOv5 and YOLOv7 algorithms all have the problem of missed detection. By analyzing the hot spot detection of the small hot spot faults in Group 3 in Figure 16, it can be seen that the SSD, YOLOv5, YOLOv7 and YOLOv8 algorithms all have the problem of missed detection, and the original unsegmented image algorithm has the problem of false detection. By analyzing the hot spot detection of the low-contrast small hot spot faults in Group 4 in Figure 16, it can be seen that the SSD, YOLOv5, YOLOv7 algorithms all have the problem of missed detection, as the weak infrared signatures are easily submerged in the background noise. By analyzing the hot spot detection in the complex environment of Group 5 in Figure 16, it can be seen that the SSD, YOLOv5, YOLOv7 and YOLOv8 algorithms all have the problem of missed detection, and the original unsegmented image algorithm has the problem of false detection.

It can be seen from the five sets of comparative experiments that when there is false highlighting feature interference in the background, the original network is prone to false detection problems. However, in this study, by constructing a split-detection serial fault detection network, the photovoltaic panel information can be highlighted to suppress the interference of redundant background on the detection network. Meanwhile, when hot spot faults present irregular small targets, traditional convolutional networks may result in some small targets not being detected. However, in this study, by introducing a DCN module into the backbone network, the shape and size of the receptive field are adaptively adjusted to be closer to the size and shape of the object, which is more robust and effectively reduces the problem of missed detections. Meanwhile, to further enhance the detection rate, the GhostNet lightweight module is introduced, which takes into account both the accuracy and real-time performance of hot spot detection.

**Figure 17:**
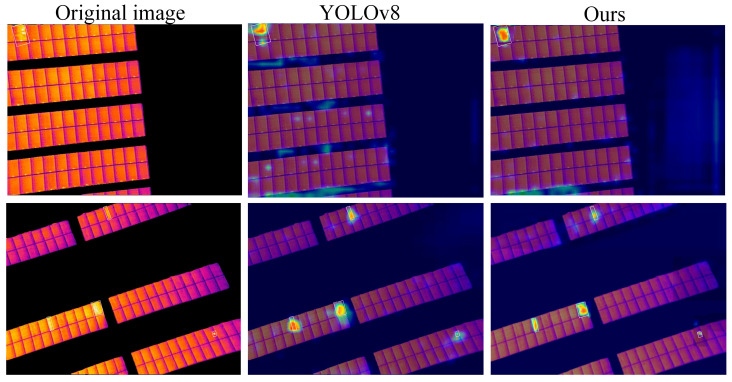
Comparison results between YOLOv8 and proposed network heat maps.

As shown in Figure 17, the attention of the reference network is often distracted by pseudo-highlight artifacts in the background, such as heated metals or reflections. In contrast, thanks to the use of heat maps for visual analysis, it is easy to more clearly and intuitively display the improvement effect of the module and highlight the important areas, as shown in Figure 10. Compared with YOLOv8, the proposed algorithm, through the regional perception of the segmentation network and the feature alignment ability of the DCN, can effectively suppress the interference of highlight artifacts and precisely focus attention on the hot spot targets within the photovoltaic panel, verifying the effectiveness of the proposed module in complex backgrounds.

To further verify the superiority of each improved part, the ablation experiments are shown in Table 3.

In Table 3, the segmentation network can extract the photovoltaic panel area from the redundant background with high significance so that the detection network can avoid the problem of false detection caused by the interference of false background highlighting. Its detection accuracy is 1.8% higher than that of the original detection network. On this basis, the DCN module has improved the detection accuracy by 2.6% by adaptively adjusting the receptive field for different objects. The small target detection head based on C2f_Ghost significantly reduces the influence of redundant gradient information on network learning through cross-stage feature fusion technology and truncated gradient flow strategy, thereby compressing the number of algorithm parameters by 48%. Ultimately, by integrating multiple improvement strategies, the mAP@0.5 index of the proposed detection algorithm can reach 88.5%, which is 10.6% higher than the detection accuracy of the original network.

To evaluate the superiority of the proposed detection network, a comparative experiment was conducted. In these experiments, all the object detection algorithms used the same data samples and kept the parameter settings unchanged. The detection results are shown in Table 4.

**Figure 18:**
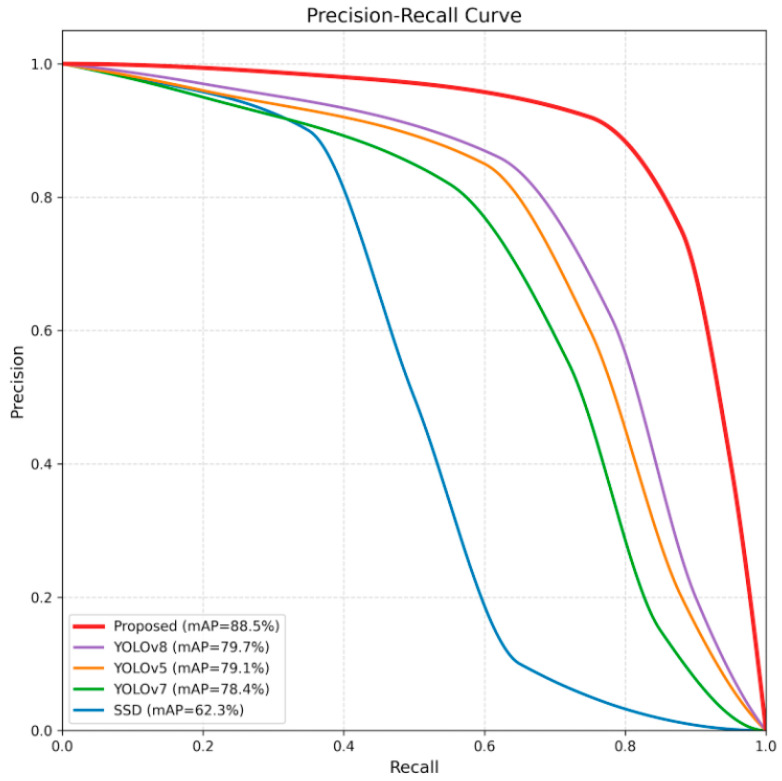
Precision/recall curves of different methods in the comparison experiments.

As can be seen from Table 4, the proposed algorithm has the highest R, mAP@0.5, mAP@0.5:0.95, and AP_small, and its P is second only to SSD. While our two-stage method introduces a segmentation overhead, the impact is managed. The average end-to-end latency is 24 ms (Segmentation: 8 ms + Detection: 14 ms + Post-processing: 2 ms) on an RTX 4060Ti. Although slightly slower than the single-stage YOLOv8 (18 ms), the significant gain in accuracy justifies the trade-off for high-precision inspection tasks. Overall, compared with the other four classic algorithms, DCN-GYOLOv8 is superior in detection accuracy and speed. In addition, Figure 18 shows the precision/recall curves of different methods, and our algorithm ranks first in detection performance.

To further evaluate the model’s robustness against varying environmental backgrounds, we conducted stratified experiments on “Mountain” (Scenario 1) and “Rooftop” (Scenario 2) datasets, as shown in Table 5. The results demonstrate that the proposed method yields superior performance in both scenarios. Specifically, it achieves an mAP@0.5 of 87.4% in the complex mountain environment and 88.9% in the rooftop scenario, outperforming the baseline YOLOv8 by 7.9% and 6.0%, respectively. This indicates that the segmentation-guided strategy effectively mitigates specific background interferences regardless of the deployment site, exhibiting strong generalization capabilities.

## 5. Conclusions

To achieve efficient inspection and defect detection of solar photovoltaic modules, this study used UAV equipped with thermal infrared cameras to collect infrared images and combined computer vision detection technology to accurately identify hot spot faults in photovoltaic modules. Considering the problems that traditional deep learning models are greatly interfered with by background noise, have a large number of parameters when detecting in the complex environment of photovoltaic power stations, and have low resolution and poor feature expression for small target detection points, a photovoltaic module hot spot fault detection method integrating U-Net and YOLOv8 was designed. The main work of this paper is as follows:

(1) Aiming at the interference of pseudo-highlighted areas with complex backgrounds on hot spot detection, the U-Net segmentation network is introduced to precisely capture the boundary features of photovoltaic panels, and its detection accuracy is improved by 1.8% compared with the original network.
(2) To address the problem of difficult detection of small targets, this study optimized the YOLOv8 network architecture by introducing deformable convolutional DCN, thereby flexibly handling the problem of insufficient receptive fields for small targets.
(3) In view of the difficulty for the detection algorithm to balance speed and accuracy, by designing the C2f_Ghost module to simplify the network parameters, the detection accuracy can be improved while ensuring the reasoning speed.

By comparing and analyzing the proposed algorithm with other classic algorithms, including the detection results before and after segmentation and image analysis, it is obvious that the proposed algorithm has the highest detection accuracy.

Despite the improved performance, the proposed method has limitations. First, the two-stage architecture increases computational cost, which may limit deployment on extremely low-power edge devices. Second, under extreme weather conditions such as heavy fog, the thermal contrast decreases, potentially affecting the segmentation quality. Furthermore, to enhance deployability in scenarios where pixel-level annotations are unavailable, we suggest a fallback workflow: the system can bypass the U-Net module and operate in a pure detection mode, accepting a trade-off of slightly higher false positive rates for lower annotation costs.

## Figures and Tables

**Table 1:** Configuration of the experimental platform.

Equipment	Configuration
System type	64-bit operating system, processor based on x64
Processor	12th Gen Intel(R) Core(TM) i5-12600KF@3.70 GHz
Graphics card	NVIDIA GeForce RTX 4060Ti
Operating system	Microsoft Windows11
Development language	Python

**Table 2:** Experimental hyperparameter settings.

Hyperparameter	Value	Description
Optimizer	SGD	Selected for stability over Adam in this task
Base Learning Rate	1 × 10^−2^	Step decay schedule applied
Momentum	0.937	-
Weight Decay	0.0005	-
Batch Size	16	-
Input Resolution	640 × 640	Standardized for all models
Total Epochs	300	Convergence typically observed around epoch 250
NMS Threshold	0.60	-
Data Split	6:2:2	Train:Validation:Test

**Table 3:** Comparison results of ablation experiments.

Segmentation Network	DCN	C2f_Ghost	mAP@0.5	mAP@0.5:0.95	Parameters
			77.9%	39.9%	3.87 M
√			79.7%	41.2%	4.95 M
√	√		82.3%	42.3%	7.31 M
√		√	80.0%	42.1%	2.37 M
√	√	√	88.5%	42.6%	5.12 M

**Table 4:** Comparison results of different detection algorithms.

Algorithms	Recall (R)	Precision (P)	mAP@0.5	mAP@0.5:0.95	AP_small	Total Latency
SSD	38.6%	88.2%	62.3%	34.5%	18.2%	0.054 s
YOLOv5	72.5%	82.2%	79.1%	40.8%	24.5%	0.027 s
YOLOv7	73.8%	72.4%	78.4%	41.2%	25.1%	0.023 s
YOLOv8	75.6%	76.2%	79.7%	42.5%	26.8%	0.018 s
Proposed	78.7%	79.8%	88.5%	49.8%	35.4%	0.014 s

**Table 5:** Performance comparison of different detection algorithms under different scenarios.

Algorithms	Scenario 1 (Mountain)			Scenario 2 (Rooftop)		
	Recall (R)	Precision (P)	mAP@0.5	Recall (R)	Precision (P)	mAP@0.5
SSD	37.7%	87.9%	60.1%	39.4%	88.5%	63.5%
YOLOv5	72.1%	83.6%	78.2%	73.6%	82.5%	80.2%
YOLOv7	72.0%	73.5%	77.9%	73.9%	73.5%	82.1%
YOLOv8	74.6%	78.2%	79.5%	75.8%	77.2%	82.9%
Proposed	76.5%	79.2%	87.4%	78.9%	79.9%	88.9%

## Data Availability

The data presented in this study are available on request from the corresponding author due to privacy.
